# We need more replication research – A case for test-retest reliability

**DOI:** 10.1007/s40037-017-0347-z

**Published:** 2017-04-07

**Authors:** Jimmie Leppink, Patricia Pérez-Fuster

**Affiliations:** 1 School of Health Professions Education, Maastricht University, Maastricht, The Netherlands; 2 Faculty of Psychology, University of Valencia, Valencia, Spain

**Keywords:** Cronbach’s alpha, Test-retest reliability, Factor analysis, Multilevel analysis, Medical education

## Abstract

Following debates in psychology on the importance of replication research, we have also started to see pleas for a more prominent role for replication research in medical education. To enable replication research, it is of paramount importance to carefully study the reliability of the instruments we use. Cronbach’s alpha has been the most widely used estimator of reliability in the field of medical education, notably as some kind of quality label of test or questionnaire scores based on multiple items or of the reliability of assessment across exam stations. However, as this narrative review outlines, Cronbach’s alpha or alternative reliability statistics may complement but not replace psychometric methods such as factor analysis. Moreover, multiple-item measurements should be preferred above single-item measurements, and when using single-item measurements, coefficients as Cronbach’s alpha should not be interpreted as indicators of the reliability of a single item when that item is administered after fundamentally different activities, such as learning tasks that differ in content. Finally, if we want to follow up on recent pleas for more replication research, we have to start studying the test-retest reliability of the instruments we use.

## What this paper adds

To follow up on recent pleas for more replication research in medical education, we have to start studying the test-retest reliability of the instruments we use. Moreover, we should refrain from interpreting Cronbach’s alpha or a similar coefficient over scores on a single item administered after different activities as an indicator of the reliability of that item and we should minimize the use of single-item measurements when multi-item instruments are available. Finally, with regard to multi-item instruments administered once in time, reliability statistics may be reported additional to but not instead of the outcomes of psychometric methods such as factor analysis.

## Introduction

The topic of replication research has been subject of discussion in journals across fields, including psychology (e. g [1–9]). and medical education [10–12], and is currently receiving a lot of attention on social media platforms such as Twitter (e. g. hashtags #replication, #replicability and #reproducibility) and LinkedIn. Since the goal of science is to establish laws and principles that have a certain general applicability, replication can be seen as one of the cornerstones of science. To enable replication research, it is of paramount importance to study the reliability of the instruments we use. That is, educators, assessors and researchers in the field of medical education largely make use of tests, questionnaires and assessments that are prone to measurement error. Without measurement error, the reliability of a measurement would be perfect (i. e. 100% or 1). In other words, the more measurement error, the lower the reliability of that measurement.

### Different aspects of reliability

Under the common convenient assumption that measurement error is random and errors of different items in a test, questionnaire or assessment tool cancel each other out, a well-known way to reduce measurement error and thus to increase the reliability of our tests, questionnaires and assessments, is to increase the number of items measuring the same variable of interest. Moreover, when human judgment is involved – such as in assessing residents’ performance in a clinical examination – one can, under that same convenient assumption of measurement errors being random and cancelling each other out, achieve a higher reliability of assessment by increasing the number of assessors. Finally, if a test, questionnaire or assessment is supposed to measure a particular variable of interest, a repeated use of that measurement instrument should yield comparable results. For instance, it would be odd to observe that an exam supposed to measure anatomy knowledge provides very different, almost uncorrelated results for the same medical students who have just completed an anatomy course and do that exam twice within an interval of a week, especially if no anatomy learning takes place between the two measurement occasions. Rather, one would expect the scores of the two measurements to correlate and the scores of the two occasions – at least for most students – to be similar. The so-called *test-retest reliability* would be higher in the case of clearly correlated measurements than for almost uncorrelated measurements.

### This article

As the psychology literature has provided reliability concepts and statistics [13–17] that have had their use in medical education research to a more or lesser extent, recent pleas for the utility of replication research in medical education [10–12] largely build forth on similar calls from the field of psychology [1–9]. Therefore, this narrative review discusses recent contributions to the reliability literature from the field of psychology and what implications these contributions have for medical education research, particularly in the context of the recent articles on the utility of replication research.

## Method

Electronic databases including Medline, PsycINFO, ERIC, Google Scholar, and Social Science Citation indices were searched using the terms *reliability, Cronbach’s alpha, test-retest reliability, factor analysis, medical education, multilevel analysis, replication, replicability* and *reproducibility*. The last three were – as hashtags (#replication, #replicability and #reproducibility) – also used for a further search of references on Twitter. Finally, all keywords served as search words in discussions on a 100,000+ members – including experts from a variety of domains – LinkedIn discussion forum *Research, Methodology, and Statistics in the Social Sciences* (https://www.linkedin.com/groups/4292855), to examine which books and articles informed discussions around reliability or replication research and to check for other potentially relevant references on these topics. Together, these search activities helped to reach convergence with regard to review or methodological articles on reliability, Cronbach’s alpha, test-retest reliability, and replication research as well as empirical research articles using Cronbach’s alpha and/or test-retest reliability, in a psychological or medical educational research context that have been cited in a variety of peer-reviewed articles and/or could help us in our endeavour to translate the insights from the psychological literature – with regard to current practices in the study of reliability and/or implications of that study for replication research – towards medical education.

## Results

A core finding from our search is an apparent mismatch between current practice in the study of reliability and what steps should be taken in the study of reliability to enable replication research. Therefore, we first review key features of current practice and their shortcomings to then present what is required for replication research.

### Key features of current practice

#### Cronbach’s alpha for a set of items measured once in time

Cronbach’s alpha [13] has been the most widely used statistic of reliability in the field of medical education [18–21], notably as some kind of quality label of test or questionnaire scores based on multiple items or of the reliability of assessment across exam stations. In this context, Cronbach’s alpha is frequently interpreted in terms of *internal consistency* or the extent to which items grouped together are indicators of the same variable of interest.

Suppose, two researchers are interested in measuring to what extent residents (1) experience the content of a particular type of patient case as complex and (2) experience difficulties in understanding the instructions around the patient case. To do so, they develop a questionnaire that comprises a set of three items for each of these two aspects (i. e. complexity and instructions). Each of the six items is to be rated by the individual student, immediately after studying a patient case, on a scale ranging from 1 (very low) to 7 (very high).

When the sample is of sufficient size, the researchers can use psychometric methods such as factor analysis on the data acquired to assess if the items can indeed be considered to form two factors as expected [22–24]. If the inter-correlation of either of the subsets of three items each is poor and/or the correlation between items from the two different subsets is as strong as or stronger than the inter-correlation within each set of items, factor analysis will prefer another solution than the expected two-factor solution. Conversely, if factor analysis supports the expected two-factor solution, we can treat the two sets of items such that they each measure two underlying variables (one by each set) that may or may not be correlated to some extent.

Sometimes, the step of factor analysis is ignored and researchers report Cronbach’s alpha over each set of items – or even one Cronbach’s alpha value for all sets of items together – straight away. Cronbach’s alpha is then usually interpreted as the extent to which the series of items – over which it is reported – measure the same underlying variable. This interpretation is counter-logical, because items under consideration measuring the same underlying variable is the very *assumption underlying Cronbach’s alpha *[13]. In other words, while Cronbach’s alpha – or one of its perhaps more viable alternatives [14, 25–27] – may provide additional information in the form of a *single *reliability coefficient for each set of items that has been indicated by factor analysis to measure the same underlying variable, it cannot serve as evidence that a given set of items actually measures the same underlying variable. In other words, Cronbach’s alpha or alternative reliability statistics may complement but not replace psychometric methods such as factor analysis.

#### Cronbach’s alpha for fundamentally different activities across time

A second situation in which Cronbach’s alpha is not rarely encountered is when researchers are interested in the reliability of a single item. An example context in which this approach has been applied in educational and psychological research during the last two decades is when dealing with students’ ratings on a scale from 1 (very, very low) to 9 (very, very high) of how much mental effort they invested [28] in each of a series of different learning tasks (i. e. one rating per learning task, e. g. [28–30]).

While estimating test-retest reliability of an item such as the one on mental effort can be useful when that item is administered repeatedly under the same or highly similar circumstances – such as with learning tasks on the same content, of very similar difficulty, and following the same structure – attempts to estimate reliability become more cumbersome when the item is administered on learning tasks that differ considerably in either of content, difficulty or structure. The reasoning behind the latter statement is similar to the one applied in the context of multiple items administered once in time: reliability statistics assume that the items under consideration measure the same underlying variable. Under that assumption, there is no variance due to measuring different variables, and distance from perfect reliability (i. e. 100% or 1) is due to measurement error. However, if some of the learning tasks about which students rate their mental effort deal with clinical knowledge whereas other learning tasks deal with statistical knowledge, two sources of variance resonate in our reliability statistics: variance due to measurement error and variance due to task differences. This is unfortunate, as reliability statistics are supposed to only indicate something about measurement error yet that cannot be separated from variance arising from tasks that vary in content, difficulty or structure.

The aforementioned does not mean that we cannot study correlations between ratings across learning tasks [31]. We should just keep in mind that – when dealing with clearly different activities over which single item ratings are collected – more than measurement error determines these correlations and, consequently, these correlations or coefficients based on them cannot really be interpreted as reliability coefficients. Moreover, even when dealing with highly similar learning tasks (e. g. same content), more difficult tasks may yield different ratings and different variation across respondents than easier tasks, and ratings from adjacent occasions (e. g. first and second task) may co-vary more or less than ratings from non-adjacent occasions (e. g. first and third task) [32]. Likewise, different items administered after a particular learning task once in time may result in somewhat different mean responses and somewhat different standard deviations around these mean responses. Cronbach’s alpha assumes that all items under consideration are on the same scale, have the same standard deviations, and that the covariance across pairs of items is the same as well [13, 15, 33]. Various scholars have advised that when these assumptions are violated, which is quite frequently the case [26], researchers should consider alternative coefficients such as the greatest lower bound (GLB) [27] or omega [14, 25], which are available in some software, including a freely available R package [26].

#### Reporting Cronbach’s alpha where test-retest reliability should be considered

A third situation in which Cronbach’s alpha is used quite frequently is when researchers fail to appreciate the potential of repeated measures data and either aggregate repeated measurements to single scores (e. g. multiple effort ratings to one average effort rating per respondent) [28, 34, 35] or multiply the number of respondents by the number of repeated measurements to obtain a ‘larger’ sample size [36]. For example, each of 52 respondents providing four effort ratings is then treated as a sample of 208 responses as if each of 208 respondents provided a single rating. This practice is also sometimes encountered in situations where a limited sample of say ten participants completed a multi-item questionnaire more than once, for instance in the aforementioned three items on perceived complexity after each of four patient cases on the same type. In this context, one Cronbach’s alpha value – perhaps preceded by a factor analysis – is then computed over the set of three items as if the responses came from forty (i. e. ten times four) respondents at a single point in time. This is unfortunate for two reasons. Firstly, the Cronbach’s alpha value may be quite different from what it should be, as it is based on a clearly wrong assumption with regard to your data (i. e. forty independent responses instead of ten sets of repeated and hence correlated responses). Secondly, this approach results in a total loss of information with regard to test-retest reliability. A more appropriate approach is found in two-level analysis which treats respondent (upper level) and measurement occasion (lower level) as hierarchical levels and provides information with regard to how scores of the repeated measurements correlate [32].

Some readers may start to wonder if Cronbach’s alpha – or perhaps one of its alternatives (i. e. GLB and omega) – calculated over a set of items administered at a single point in time can provide information with regard to test-retest reliability. The answer is ‘no’. Suppose, residents are tired of participating in your study and individually decide to randomly pick a number on the 0–10 scale and rate all three items on perceived complexity of a patient case with that number (e. g. one participant rates 7‑7-7, the next one 4‑4-4, etcetera). Half an hour later, they are told that they need to respond to these questions again. Again, they randomly pick a number (probably a different one than the first time) and rate all three items with that number. Given different numbers across participants but no variation within participant, Cronbach’s alpha would be exactly one for each occasion. However, test-retest reliability could well be close to zero in this case. Judging from the Cronbach’s alpha, one might think that we are dealing with very reliable measurements; once taking a look at the test-retest reliability, things look less positive. Finally, in this context, it is worth mentioning that lower Cronbach’s alpha values do not necessarily always imply lower test-retest reliability; one can obtain acceptable test-retest reliability values even when Cronbach’s alpha values are on the lower side.

### Needed to enable replication

#### Reporting on the reliability of sets of items administered once in time

The fact that Cronbach’s alpha and other reliability statistics used for reporting on the reliability of sets of items administered at one point in time tell us close to nothing about test-retest reliability pertaining to the repeated administration of the same sets of items does not mean that the reliability of sets of items administered once in time is a topic that no longer has any importance. We just need to keep in mind that Cronbach’s alpha or alternative reliability estimates may complement but not replace psychometric methods such as factor analysis [15, 22–24]. When replicating an initial study which used a questionnaire that resulted in an anticipated two-factor solution, the replication study should preferably also include a factor analysis to examine whether the same sets of items can be grouped together (i. e. more or less the same factor structure). If factor analysis fails to replicate the two-factor structure from the initial study, that means we have insufficient ground to assume that the two studies measure the same two underlying variables consistently. Reliability estimates such as Cronbach’s alpha cannot reasonably be expected to provide us with information on that. In other words, providing factor loadings and cross-loadings for each item is more useful than providing single reliability coefficients. Moreover, given the development of reliability coefficients – notably GLB and omega [14, 25–27, 33] – that appear to better account for the data features than Cronbach’s alpha under a variety of realistic circumstances and the increasing availability of these coefficients in statistical packages (e. g. [26]), we may want to consider reporting multiple coefficients [14] if not report these newly developed coefficients instead of Cronbach’s alpha [26, 27].

#### Do not use single-item measurements when multi-item instruments are available

Apart from the limitations of Cronbach’s alpha and related coefficients in the context of multi-item measurements discussed in the previous paragraph, such coefficients should not be interpreted as indicators of the reliability of a single item when that item is administered after fundamentally different activities, such as learning tasks that differ in content.

In fact, single-item measurements are – from a methodological point of view – very hard to defend. If we do not have reasons to believe that an experiment with a single participant is a strong experiment (i. e. we do not believe in *le cas pur*), why would we go with single-item measurements (i. e. to believe in *la question pure*) if we can develop and use multi-item measurement instruments instead? One of the first arguments of this article was that a well-known way to reduce measurement error and thus to increase the reliability of our instruments is to have more items measuring the same variable of interest. The use of single-item measurements is to be minimised – and, in the presence of multi-item instruments even better, avoided – for a number of reasons. Firstly, neither psychometric methods such as factor analysis nor reliability coefficients can be used to examine the reliability of measurement at a given point in time. This is problematic especially because single-item measurements are typically more noisy than multi-item measurements. Any attempts to interpret Cronbach’s alpha of multiple measurements with the same item in terms of internal consistency or even test-retest reliability fail when this item is administered over different activities such as clearly different learning tasks, because two sources of variance – measurement error and variance due to task differences – are perfectly confounded [32]. Secondly, single items cannot distinguish between multiple variables of interest. Let us demonstrate this with an example: mental effort ratings [28].

For more than two decades, participants’ mental effort ratings have been assumed to reflect overall cognitive load as a combination of different sources of cognitive load [28, 30]. Different participants may experience each source of cognitive load to a different extent [37]. Hence, differences in mental effort ratings may reflect differences in either source of cognitive load or both – if not measurement error only – but we cannot tell which source of cognitive load is varying to what extent. Single items can, apart from measurement error, capture one source of variance at best [32]. Although experimental manipulations may create groups of participants that, on average, are comparable in terms of a particular source of cognitive load, there may still be differences between participants in the same group in that source of cognitive load. Hence, we can never tell whether a difference in mental effort reflects a difference in one type of cognitive load or another. This is unfortunate, because when we correlate mental effort ratings with for instance learning outcome measurements, we cannot really tell what the correlation means. Add to this that, using multi-item cognitive load instruments, recent studies indicate that mental effort ratings may at best correlate with one particular source of cognitive load but fail to capture the other source(s) of cognitive load [38–40], and we have a solid case for preferring multi-item above single-item cognitive load measurements.

In sum, a methodologically solid empirical study takes neither *le cas pur* nor *la question pure* as starting point: we should include a sufficient number of participants in our studies and we should prefer multi-item measurements above single-item measurements. Multi-item instruments enable factor analysis and enable replication studies that include factor analysis to determine if the same sets of items can reasonably be grouped together in different (i. e. initial and replication) studies.

#### An appropriate interval of measurements for test-retest reliability

When an instrument is supposed to measure a particular underlying variable, such as the aforementioned perceived complexity or usefulness of instruction around a particular type of patient case, you would expect that repeated administrations of that instrument yield similar scores for the same respondents unless the variable you measure with the instrument is subject to change from one occasion to the next. This question of test-retest reliability is of paramount importance to replication research [11]: without decent test-retest reliability, administering an instrument on a Tuesday versus on a Thursday could make a big difference, and any attempts to replicate findings from an initial study using that instrument could be considered a waste of time.

To meaningfully estimate test-retest reliability, we need repeated measurements administered in appropriate time intervals [41, 42]. Although this approach may be difficult to implement in educational practice, instrument development and experimental studies can include a repeated measurements component. The length of the interval should be such that it is long enough that memory or practice effects can fade and at the same time is too short for maturational or historical changes to occur on the part of the respondent. For example, when interested in the test-retest reliability of a questionnaire of six or more items on cognitive load experienced in a learning period that just finished [32, 37, 38], an interval of ten to fifteen minutes may do. Moreover, waiting much longer in that case – for instance a day – may introduce bias due to forgetting. However, in the context of an adult vocational interest inventory [15], for instance, where adults may remember some of their responses to test items over a sustained period and interest may be stable over a long time span, a test-retest interval of six months to two years might be more appropriate. Finally, randomized controlled experiments in which participants learn something that is then assessed through a post-test sometimes also include a follow-up test that is administered a week or a couple of weeks later. An interval of a day might not be sensible due to memory or practice effects, whereas an interval of several months could come at an increased risk of maturation (i. e. learning during the interval). In short, the length of an interval depends on the purpose and context of the study and may influence to some extent the outcomes of test-retest reliability. However, when we define reliability as the extent to which repeated administration of the same instrument among the same respondents yields similar results, we need test-retest reliability to address that question.

#### Reporting on the test-retest reliability of our instruments

To estimate test-retest reliability, we can use the same methods as are commonly encountered in the context of inter-rater reliability: Cohen’s kappa for dichotomous or polytomous (i. e. three or more categories) response variables [16], weighted kappa statistics for polytomous ordinal response variables [17], Pearson’s correlation coefficient *r* for non-categorical (i. e. scale) response variables [23, 24], and the intra-class correlation coefficient (ICC) for both categorical and non-categorical variables [43–46]. Where kappa and *r* can be used when dealing with two measurements (two time points or two raters), the ICC can be used with more than two measurements as well. Moreover, while *r* indicates to what extent scores of a quantitative variable measured at two measurements correlate, the ICC can combine an estimate of correlation with a test in the difference in mean scores of the measurements [43, 47]. That is, differences in means do not affect *r* but do lower the ICC to some extent. If a researcher’s interest is solely in the stability of scores (i. e. same or similar position of respondents’ scores towards each other at two measurements, regardless of the mean scores of the measurements), *r* can provide an indication of that stability. However, if one wishes to incorporate mean differences in the reliability estimate as well (i. e. a penalty for large differences in mean scores across measurements) one needs to consider specific models that provide an ICC [43–47]. Finally, in the context of factor analysis and related methods for latent variable analysis, one can consider including a time component in the model when dealing with repeated measurements [22], which allows one to simultaneously examine whether a factor structure (i. e. sets of items grouped together) is stable across measurements and obtains information with regard to the correlation between factor scores of the different measurements (i. e. test-retest reliability).

## Discussion

Following debates in psychology on the importance of replication research [1–9], we have also started to see calls for a more prominent role for replication research in medical education [10–12]. To enable replication research, it is of great importance to carefully study the reliability of the instruments we use. Cronbach’s alpha has been the most widely used estimator of reliability in the field of medical education, notably as some kind of quality label of test or questionnaire scores based on multiple items or of the reliability of assessment across exam stations. However, as this narrative review outlines, Cronbach’s alpha or alternative reliability statistics may complement but not replace psychometric methods, such as factor analysis. Moreover, multiple-item measurements should be preferred above single-item measurements, and when using single-item measurements, coefficients such as Cronbach’s alpha should not be interpreted as indicators of the reliability of a single item when that item is administered after fundamentally different activities such as learning tasks that differ in content, difficulty or structure. Finally, if we want to follow up on the recent pleas for more replication research, we will have to start studying the test-retest reliability of the instruments we use. Although the latter does require additional planning in the design of studies on the development of psychometric instruments and the design of experiments, test-retest reliability is the only way to provide us with an indication of the extent to which repeated administration of the same instrument among the same respondents yields similar results. Reliability coefficients calculated over sets of items measured once in time may have their use complementary to, for instance, factor analysis but cannot provide us with information about test-retest reliability.

In sum, to enable meaningful replication research – which is an inherent part of science – careful study of the reliability of the instruments we use is needed. We fully support the recent pleas for more replication research as well as efforts by journals such as this one to give more opportunities to researchers for replication studies, and hope that we have provided useful guidelines to facilitate the analysis of the reliability of the instruments used in medical education research in order to enable meaningful replication research.
